# Influence of clearance on the time-dependent performance of the hip following hemiarthroplasty: A finite element study with biphasic acetabular cartilage properties

**DOI:** 10.1016/j.medengphy.2014.05.014

**Published:** 2014-11

**Authors:** Junyan Li, Xijin Hua, Zhongmin Jin, John Fisher, Ruth K. Wilcox

**Affiliations:** a Institute of Medical and Biological Engineering, School of Mechanical Engineering, University of Leeds, UK; b School of Mechanical Engineering, Xi’an Jiaotong University, China

**Keywords:** Hip, Hemiarthroplasty, Contact mechanics, Articular cartilage, Biphasic, Finite element

## Abstract

Hip hemiarthroplasty is a common treatment for femoral neck fracture. However, the acetabular cartilage may degenerate after hemiarthroplasty leading to postoperative failure and the need for revision surgery. The clearance between the acetabular cartilage and head of the prosthesis is one of the potential reasons for this failure. In this study, the influence of joint clearance on the biomechanical function of a generic hip model in hemiarthroplasty was investigated using biphasic numerical simulation. Both a prolonged loading period of 4000 s and dynamic gait load of 10 cycles were considered. It was found that a larger clearance led to a higher stress level, a faster reduction in load supported by the fluid and a faster cartilage consolidation process. Additionally, the mechanical performance of the acetabular cartilage in the natural model was similar to that in the hemiarthroplasty model with no clearance but different from the hemiarthroplasty models with clearances of 0.5 mm and larger. The results demonstrated that a larger clearance in hip hemiarthroplasty is more harmful to the acetabular cartilage and prosthesis heads with more available dimensions (i.e. smaller increments in diameter) could be manufactured for surgeons to achieve a lower clearance, and reduced contact stress in hemiarthroplasty surgeries.

## 1 Introduction

Hip hemiarthroplasty, a surgical procedure in which the femoral head is replaced by a metallic prosthesis, is a common treatment option for joint degradation that only affects the femoral head (e.g. femoral neck fracture). Although it is less destructive, less costly and requires shorter surgical time than a total hip replacement procedure, the acetabular cartilage, when articulating with a metallic head component, may degenerate, resulting in pain, immobility and the need of a revision surgery [1], [2], [3]. Therefore maintaining the well-being of the acetabular cartilage in hip hemiarthroplasty is important for the long-term performance of the joint.

Selection of the femoral component size is crucial for hip hemiarthroplasty, as it is directly linked with acetabular function and degeneration [3], [4]. Empirically, surgeons initially use a head template of various dimensions to determine the size of the acetabulum and then adopt the largest prosthesis that is smaller than the template to achieve the smallest clearance between the prosthesis and acetabulum. However, artificial heads are most frequently available with 2 mm increments in diameter, which still leads to a mismatch in curvatures. A small head with larger clearance can lead to reduced joint conformity, lower stability, increased stresses and a faster cartilage consolidation process, while a large head may increase the periacetabular stresses and the coefficient of friction [4], [5], [6]. The interaction between the femoral component size and joint performance is, as yet, poorly defined. A greater understanding of this relationship could provide insight into whether the current surgical options are adequate and provide guidelines on what dimension of the prosthesis to adopt in order to improve the outcome of hip hemiarthroplasty surgery.

Mechanical factors have long been recognised as the primary contributor to cartilage damage. The function and degeneration of cartilage is closely linked with its biphasic (i.e. fluid–solid) nature, because the fluid phase is able to support most of the compressive load applied to the tissue and it also provides an excellent lubrication environment [7], [8], [9]. Particularly for the natural hip joint which is highly conforming, the fluid can provide over 90% load support for a prolonged period, as found recently by the authors using a novel biphasic computational model [10], [34]. It is therefore necessary to consider the cartilage as a biphasic structure to obtain a greater understanding of the function and degeneration mechanisms of the hip joint following hemiarthroplasty.

The time-dependent tribological performance of the hip following hemiarthroplasty has been measured experimentally by Lizhang et al. [4], which is most likely associated with the fluid in the cartilage, supporting the importance of a biphasic investigation. However, the fluid pressure distribution within the hip cannot be determined through current experimental techniques, and a computational approach serves as the only method by which the biphasic behaviour of the joint can be fully investigated. Using a biphasic finite element (FE) simulation, Pawaskar et al. [11] evaluated the mechanical response of a hemiarthroplasty hip model for a variety of activities over short periods. However, the effect of different head sizes (joint clearances) and a prolonged loading period on the biphasic behaviour of hip in hemiarthroplasty has not been investigated, due to the limitations of that biphasic model. The aim of this study was therefore to use a biphasic FE model to evaluate the influence of joint clearance on the biphasic performance of the hip joint following hemiarthroplasty during a prolonged physiological loading period and under a dynamic load representing the gait cycle.

## 2 Methods

**Fig. 1:**
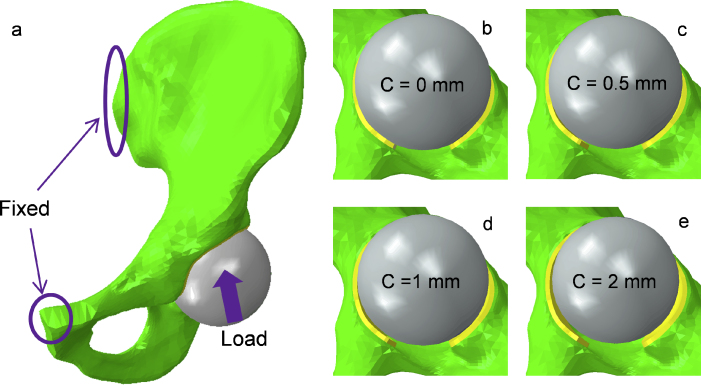
The three dimensional hip model in hemiarthroplasty (a) and the metal heads with four different dimensions articulating against with the acetabular cartilage (b–e) (C: clearance).

The hip hemiarthroplasty FE model used in this study was composed of a pelvis with the acetabular cartilage in articulation with a metallic prosthetic head component (Fig. 1). The labrum was not considered in this study, since it is commonly incomplete after hip hemiarthroplasty surgery. Details of model construction for the pelvis and acetabular cartilage were described in a previous study [10]. Briefly, the acetabular cartilage was assumed to be spherical (radius = 28 mm) with a uniform thickness of 2 mm to create a generic geometry for the acetabulum. The bone was represented by around 91,600 tetrahedral elements and the cartilage was meshed with 8400 hexahedral elements. A sensitivity study on the number of elements was conducted to ensure the model was insensitive to a denser mesh. The cartilage and bone were bound together through sharing the same nodes on their interface. The cartilage was assumed to be biphasic, whereby the solid phase was represented as neo-Hookean material (aggregate Young's modulus *E* = 1.2 MPa, Poisson's ratio *ν* = 0.045) with a constant permeability (*K* = 0.0009 mm^4^/N s) [12]. The bone was modelled as impermeable and linearly elastic with Young's modulus of 17,000 MPa and Poisson's ratio of 0.3 [13]. The cortical bone and trabecular bone were not modelled separately because they were found to have little influence on the model predictions of interest for this study [10].

The metallic head component was represented by a rigid and impermeable sphere. To evaluate the influence of head size on the model predictions, four different radial clearances (0 mm, 0.5 mm, 1 mm and 2 mm) were evaluated by varying the size of the head (Fig. 1). The contact between articulating surfaces was assumed to be frictionless due to the low friction coefficient [14], [15]. The fluid flow on the articulating surfaces was defined as contact-dependent so that fluid exudation was prevented on the cartilage surface that was in contact with the impermeable head but allowed for open surfaces. The pelvis was fixed at the sacroiliac and pubis symphysis joints. Loads were applied to the centre of the metallic head which was fixed in rotational degrees of freedom but allowed to move translationally for self-alignment. Rotation of the head was not considered because of its spherical geometry and the frictionless assumption of the articulating surfaces. Two common kinds of loads were considered: (1) a static load of approximately 2130 N based on the average data for one leg stance, ramped over 0.6 s and then held constant for 4000 s; (2) a time-dependent dynamic load during 10 cycles of gait – this load varied in magnitude and direction through each cycle to represent walking at normal speed (1.1 m/s) [16]. Additionally, the natural whole joint model with a radial clearance of 0.5 mm as described in a previous study [10] was considered for comparison.

The modelling procedure has been previously validated by comparing the model predictions to experimental tests, and good agreement in contact mechanics was achieved [17], [35]. FE analyses were conducted using the open-source solver FEBio (version 1.5.0; Musculoskeletal Research Laboratories, Salt Lake City, UT, USA; URL: mrl.sci.utah.edu/software/febio) [18] owing to its good convergence capability in the simulation of biphasic materials in contact [10]. Contact stress, contact area, fluid pressure and fluid support ratio (the load supported by the fluid pressure over the total load) were recorded.

## 3 Results

**Fig. 2:**
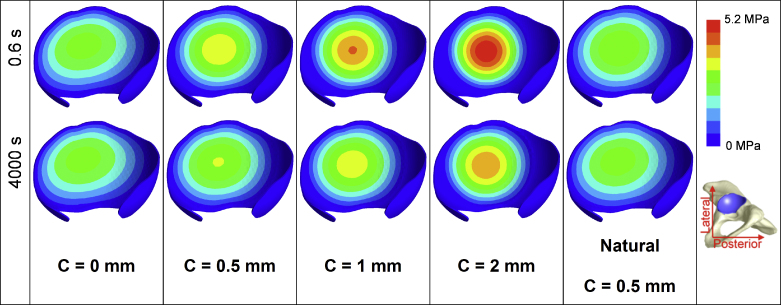
Contour of the contact stress (MPa) of the acetabular cartilage of the natural hip model and the hip models in hemiarthroplasty with different head dimensions at 0.6 s and 4000 s in the static loading case (lateral and posterior refer to the orientation of the pelvis during standing).

Contours of contact stress for all the models under the static load at 0.6 s and 4000 s are presented in Fig. 2. The hemiarthroplasty model with larger clearance had a higher peak contact stress, a faster cartilage consolidation process as evidenced by the greater changes in the stress distribution, and a smaller contact area that was concentrated within the central region of the cartilage surface. Additionally, the contour of contact stress for the natural hip model was similar to the hemiarthroplasty model with no clearance both at 0.6 s and 4000 s.

**Fig. 3:**
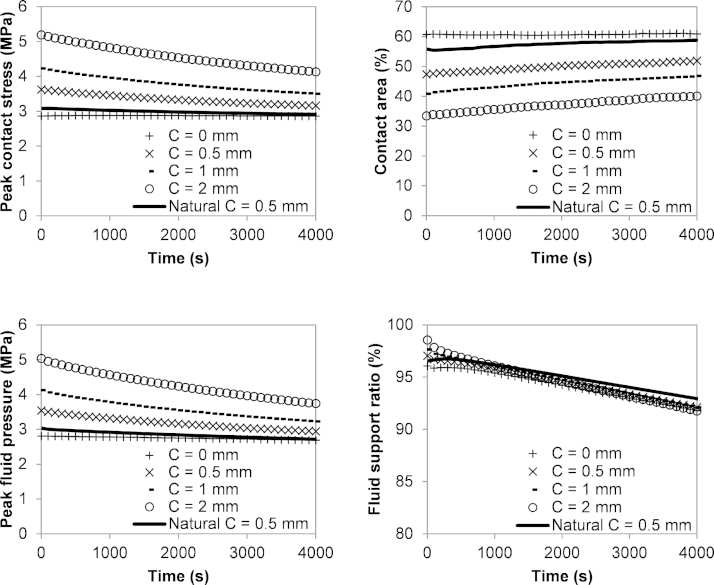
Results over 4000 s period for the natural hip model and the hip models in hemiarthroplasty with different head dimensions in the static loading case.

Results of the models under the static load over 4000 s period are summarised in Fig. 3. The hemiarthroplasty model with larger clearance had higher peak contact stress, higher peak fluid pressure, smaller contact area and greater changes in these results over the 4000 s. The peak contact stress decreased by 20.5% for the hemiarthroplasty model with 2 mm clearance, while there was almost no change (<1%) in the peak contact stress for the model with no clearance over the 4000 s period. The fluid support ratio was above 90% over the 4000 s period for all the models, and was slightly higher but decreased faster for models with larger clearances. The decrease in fluid support ratio was 6.9% and 4.5% for the model with a clearance of 2 mm and 0 mm, respectively. Comparable results were found between the natural hip model and the hemiarthroplasty model with no clearance.

**Fig. 4:**
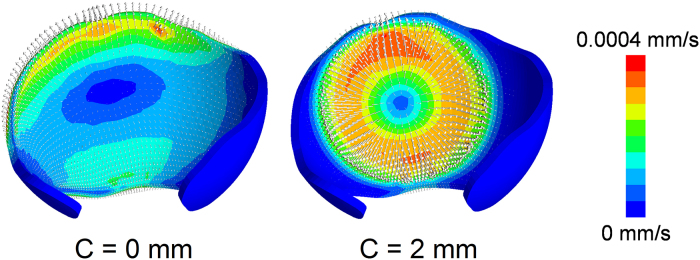
Contour of fluid flux on the acetabular cartilage for the hemiarthroplasty model with 0 and 2 mm radial clearances respectively at the instantaneous period in the static loading case. The directions of fluid flux were exhibited by grey vectors.

The fluid flux for the hemiarthroplasty models with 0 mm and 2 mm clearances is illustrated in Fig. 4. For the model with no clearance, fluid flux mainly occurred around the edge region of the cartilage. For the model with 2 mm clearance, however, the fluid flux was higher in magnitude and scattered across a larger area around the central region of the acetabular cartilage, suggesting a faster consolidation process.

**Fig. 5:**
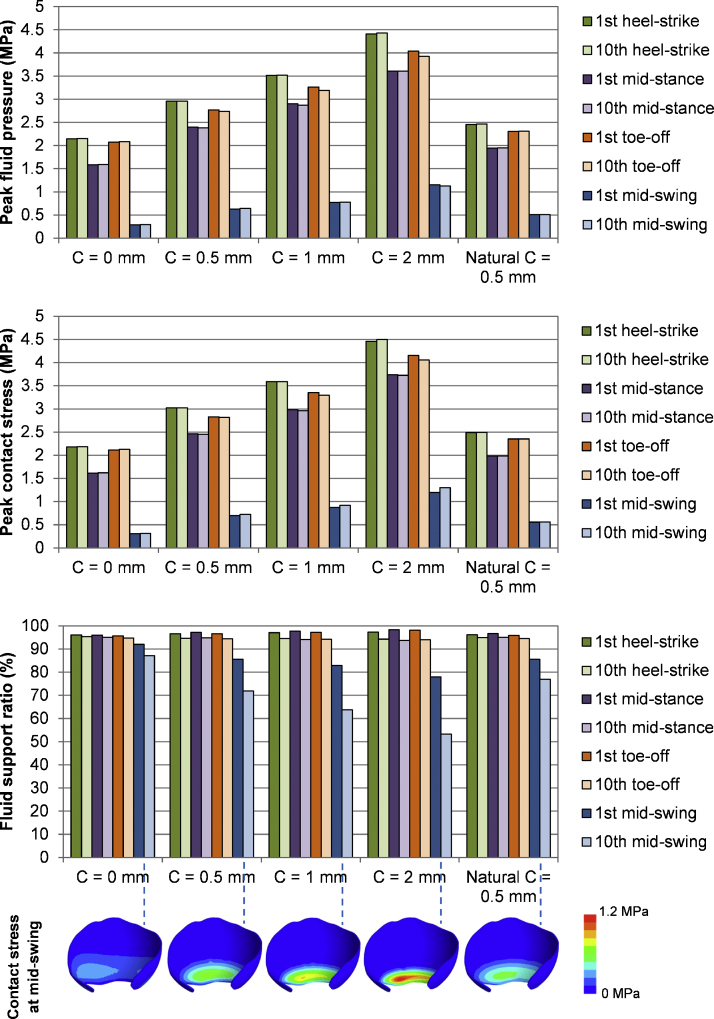
Results of the natural hip model and the hip model in hemiarthroplasty with different head dimensions over 10 cycles of gait, along with the contours of contact stress at mid-swing during the 1st cycle.

A summary of results for the gait loading case is presented in Fig. 5. Again, similar results were found between the natural hip model and the hemiarthroplasty model with no clearance. Higher peak contact stress and peak fluid pressure were observed for the hemiarthroplasty model with larger radial clearance. Very little change in the peak contact stress and peak fluid pressure was detected over 10 cycles. However, an obvious drop in fluid support ratio over the cycles was found for the model with larger clearance, particularly during the mid-swing phase when contact occurred at the interior region of the acetabular cartilage. For the model with a clearance of 2 mm at the mid-swing phase, the fluid support ratio was 78% at 0.6 s and decreased to 53% after 10 cycles of gait. Greater contact concentration around the interior edge of the acetabular cartilage was found in the model with larger clearance during mid-swing.

## 4 Discussion

In this study, the influence of joint clearance on the biphasic performance of the hip following hemiarthroplasty was investigated using a FE model. Both a static load during a prolonged loading period and a dynamic gait load over several cycles were considered in order to simulate the circumstances commonly experienced by the hip. A similar investigation was conducted by Pawaskar [19] in which a biphasic model of the hip following hemiarthroplasty was used to examine the performance of the treatment with varying clearances under a static load for 600 s. The major limitation of that study was that the time-dependent joint response was not evident over the loading period of 600 s, thus limited information on joint performance or likelihood of degeneration could be derived. A longer loading period was not achieved due to convergence difficulties with the model. By adopting a newly developed modelling technique [10], in the current study, the loading period was extended to an extreme period of 4000 s for loads of physiological magnitude, whereby an obvious cartilage consolidation process was detected. Only 10 cycles of gait were evaluated here because the small time step that is necessary to represent variation in load over the cycle required a lengthy simulation period. Yet, the consolidation process of the joint over 10 cycles of gait was detected, providing a pattern that can be used to predict the trend for more cycles.

During the early loading period, a larger clearance leads to a smaller contact area, substantially increased peak contact stress, but a slightly lower fluid support ratio, which would contribute to a higher stress level in the solid matrix of the acetabular cartilage, a greater level of friction and a greater potential to degenerate. Over a prolonged loading period, the cartilage consolidated faster in the model with larger clearance, because of the faster fluid exudation, also suggesting a more harmful mechanism with a large clearance.

Over 10 cycles of gait, an increased peak contact stress and faster cartilage consolidation process were also observed for the model with larger clearance. In particular at mid-swing, the fluid support ratio was lower and decreased substantially faster in the model with larger clearance when contact occurred near the interior edge of the acetabular cartilage. This is because the tissue around the interior region of the acetabular cartilage was less confined and thus had a lower capability to support fluid than the tissue in the central acetabular cartilage [10], [20], [21], and at the same time, the interstitial fluid can easily exudate from the interior edge. The greater contact concentration associated with the model with a larger clearance, as evidenced by a smaller contact area and higher peak stress, may lead to a greater proportion of load being transferred to the solid matrix and a faster consolidation process for the tissue around the edge region. Besides, a lower fluid support ratio means a higher portion of load shared by the solid matrix, suggestive of an increased friction coefficient [22], [23]. Therefore, a larger clearance in hip hemiarthroplasty may also have a worse effect on the acetabular cartilage, particularly for the tissue around the interior region during dynamic loads.

In most of the previous numerical studies on the hip, the cartilage was assumed to be incompressible and monophasic (e.g. hyperelastic) to simulate the biphasic response of the joint during the early period of loading. As shown in the static loading case, it takes more than 1000 s to observe an obvious cartilage consolidation when the contact occurs around the central acetabulum region. However, for the time at which the contact slides toward the edge region, cartilage consolidation becomes obvious only over 10 cycles of gait (∼10 s). This suggests that monophasic joint simulations are appropriate for very specific circumstances. On the other hand, in the dynamic loading case, the pattern of fluid exudation is subject to the variation in the loading magnitude and loading direction over time, suggesting that dynamic loads should be applied in a time-dependent way for biphasic simulations.

The main limitations of this study are the adoption of a generic joint geometry and the assumption of a linear elastic solid phase for the cartilage. The linear elastic solid phase assumed in this study is not able to represent the inhomogeneous fibre-reinforced structure where the tensile stiffness is higher than the aggregate stiffness [24], [25], [26]. This assumption potentially results in underestimated peak contact stress, fluid support ratio and confinement effect of the tissue, particularly for the acetabulum edge region around which the contact occurred during mid-swing [10]. The generic joint geometry, as represented by the spherical acetabulum with uniform cartilage thickness, potentially leads to an underestimated peak contact stress and an altered shape in contact area [27]. The peak contact stress of the models in this study ranged from 3 MPa to 5 MPa, which is lower than previous experimentally measured results (i.e. 4–10 MPa) [28], [29], [30], [31], but consistent with previous numerical models with similar geometrical assumptions (i.e. 3–4 MPa) [11], [32], [33]. However, these assumptions are appropriate for the purpose of this parametric study on a generic hip, and necessary to offset the potential influences caused by individual variations. Additionally, the higher peak contact stress and faster cartilage consolidation process associated with a larger clearance, as observed in this study, are well supported by a recent in vitro experimental study [4] using porcine hips following hemiarthroplasty. This also suggests that the models used here, although with several simplifications, are able to accurately capture the cause-and-effect relationship for parametric analysis purposes.

In both the dynamic loading and prolonged static loading cases, a larger clearance of the hip in hemiarthroplasty was found to be more harmful to the acetabular cartilage, as evidenced by a higher stress level and faster cartilage consolidation process. The biomechanical function of the acetabular cartilage in the natural model was similar to that in the hemiarthroplasty model with no clearance but different from the hemiarthroplasty models with clearances of 0.5 mm and larger, suggesting that clearance needs to be avoided or minimised to ensure the joint following hemiarthroplasty is as close to the normal healthy mechanical environment. It is also recommended that prosthesis heads with more available dimensions (i.e. smaller increments in diameter) should be manufactured for surgeons to achieve a minimal clearance during hemiarthroplasty surgery. Further studies will focus on subject-specific evaluations to provide more stratified intervention guidelines.
